# The impact of *Staphylococcus saprophyticus* on the fermentation of cigar filler tobacco leaves and the dynamics of microbial community

**DOI:** 10.3389/fbioe.2025.1666879

**Published:** 2025-09-23

**Authors:** Lan Yao, Zhongde Zhao, Linwei Li, Jun Yu, Jinpeng Yang, Chunlei Yang, Xiong Chen

**Affiliations:** ^1^ Key Laboratory of Fermentation Engineering (Ministry of Education), Cooperative Innovation Center of Industrial Fermentation (Ministry of Education & Hubei Province), College of Life and Health Science, Hubei University of Technology, Wuhan, China; ^2^ Tobacco Research Institute of Hubei Province, Wuhan, China

**Keywords:** cigar filler leaves, *Staphylococcus saprophyticus*, microbial community, volatile aromatic compounds, functional gene analysis

## Abstract

**Introduction:**

*Staphylococcus* has garnered increasing attention for its role in improving fermentation results and promoting the biosynthesis of aromatic compounds.

**Methods:**

This study investigated the effects of exogenously introduced *Staphylococcus saprophyticus* on the microbial community structure, functional gene expression, and volatile aroma profiles during the fermentation of cigar filler leaves.

**Results:**

The results demonstrated that *S. saprophyticus* significantly enhanced the accumulation of alcohols and ketones. LEfSe analysis identified *Bacillus* as a key differential genus in the inoculated group. Spearman correlation analysis revealed positive associations between *Staphylococcus* and *Bacillus*, as well as with key aroma compounds such as 1-methyl-4-(2-methyloxiranyl)-7-oxabicyclo [4.1.0] heptane and cis-6-nonenal. EGGNOG functional annotation indicated upregulation of carbohydrate and amino acid metabolism pathways. Additionally, CAZy analysis revealed increased abundance of glycosyltransferases and carbohydrate-binding modules, which may facilitate sugar conversion and utilization.

**Discussion:**

These findings provide a theoretical basis for the application of exogenous microorganisms in cigar fermentation and offer insights into the regulation of microbial community structure for quality improvement.

## 1 Introduction

Cigars comprise multiple layers of tobacco leaves, each with distinct functions; the wrapper and filler are among the most critical. These two components differ significantly in their fermentation requirements and in their contributions to the final quality of the cigar (Zhang et al., 2024). The wrapper, as the outermost layer, primarily ensures the cigar’s aesthetic appeal and structural integrity (Ning, 2021). In contrast, the filler leaves form the core of cigar and play a decisive role in shaping its primary aroma and flavor profile. Their fermentation process is more complex, aiming to release a diverse and rich array of volatile aromatic compounds through deep fermentation, thereby defining the cigar’s sensory experience and overall quality (Yao et al., 2023).

Recent evidence suggests that introducing exogenous microorganisms can effectively regulate the fermentation process and enhance cigar quality. For example, inoculation with *Acinetobacter* has been shown to significantly increase the levels of specific volatile flavor compounds, while simultaneously reducing off-flavors and harshness, thus improving the richness and smoothness of tobacco leaves (Zheng et al., 2022). Similarly, the addition of *Bacillus altitudinis* has been reported to increase microbial richness and metabolic diversity, thereby improving fermentation efficiency and strengthening the dominance of *Bacillus* and *Corynebacterium*, which are known to play pivotal roles in the biosynthesis of aromatic compounds (Song et al., 2024). Furthermore, the introduction of *Saccharomyces cerevisiae* can refine the fermentation process of cigar tobacco leaves by promoting the generation of key aromatic compounds-particularly alcohols and esters-ultimately enhancing both the aroma complexity and sensory quality of the final product (Yao et al., 2022).

The chemical composition of filler leaves is complex, necessitating not only the generation of aromatic compounds but also deep fermentation to promote the degradation and transformation of internal constituents (Zheng, 2022). The generation and release of volatile aromatic compounds are especially crucial in filler fermentation, as these compounds directly influence the complexity and richness of cigar flavor (Chen, 2019). Thus, the role of exogenous microorganisms in filler leaves fermentation extends beyond simple aroma generation.

*Staphylococcus saprophyticus*, a biosafety-level-1 (BSL-1) microorganism ubiquitous in natural environments and therefore considered low-risk, has demonstrated substantial potential in food fermentation and environmental adaptation (ATCC 15305). Previous studies have shown that the protease and lipase activities of *Staphylococcus saprophyticus* can synergistically catalyze the release of flavor precursors, thereby promoting the formation of key aroma compounds such as aldehydes and ketones (Wang et al., 2021). The deep fermentation of cigar filler leaves imposes higher demands on microbial metabolic regulation, requiring the targeted degradation of intrinsic compounds such as sugars, proteins, and esters to drive efficient production and accumulation of aromatic substances. Preliminary results have demonstrated that *S. saprophyticus* is capable of stable growth under non-saline conditions and exhibits a certain capacity to enhance aroma formation. Moreover, this strain can modulate key metabolic pathways, particularly those involved in amino acid metabolism, thereby facilitating the biosynthesis of aromatic compounds (Jo et al., 2022), which aligns closely with the core objective of cigar tobacco fermentation.

This study aims to systematically investigate the application of *Staphylococcus saprophyticus* in the fermentation of cigar filler leaves, with a particular focus on its influence on microbial community structure, volatile aroma compound accumulation, and key metabolic pathways. Through metagenomic analysis, this research could provide a theoretical basis and practical reference for improving fermentation processes and enhancing product quality in cigar industry.

## 2 Materials and methods

### 2.1 Materials

CX-14 filler leaves were harvested in 2023 from the Enshi region of Hubei Province and were provided by the Cigar Tobacco Leaf Production Base in Enshi. *Staphylococcus saprophyticus* was isolated from the surface of cured meat and preserved in glycerol tubes at −80 °C. LB medium was composed of 10 g/L tryptone, 5 g/L yeast extract, and 10 g/L sodium chloride, adjusted to a pH of 7.2–7.4, and sterilized at 121 °C for 20 min.

### 2.2 Methods

#### 2.2.1 Preparation of *Staphylococcus* culture

One milliliter of preserved *Staphylococcus saprophyticus* culture was inoculated into liquid LB medium and incubated at 200 rpm for 12 h to prepare the seed culture. This seed culture was subsequently transferred into a fermenter under the following conditions: natural pH, aeration rate of 1 m^3^/h, and agitation at 400 rpm. After 12 h, the culture was centrifuged, and the resulting pellet was washed three times with distilled water and stored at 4 °C for further use.

#### 2.2.2 Box fermentation of cigar filler leaves

In the control group (CK), only water was added, while in the experimental group (J), *Staphylococcus saprophyticus* was added at 1.3% of cigar filler leaves weight. Both groups were re-moistened to moisture content of 34% ± 2%. Samples were collected in triplicate at four time points: before fermentation (CK-0, J-0), after the first overturn (CK-1, J-1; day 7), after the second overturn (CK-2, J-2; day 14), and after the third overturn (CK-3, J-3; day 35). All subsequent analyses were conducted using these biological replicate samples. The entire fermentation process lasted 35 days under controlled conditions (28 °C ± 2 °C, relative humidity 80% ± 2%). After fermentation, the leaves were dried at 75 °C, ground, and passed through a 60-mesh sieve.

#### 2.2.3 Simultaneous Distillation Extraction (SDE) for determining neutral aroma compounds

Ten grams of sieved sample were placed in a 1,000 mL round-bottom flask, to which 200 mL of saturated saline solution was added. In parallel, 60 mL of dichloromethane was added to a 100 mL round-bottom flask. Both flasks were connected to the Simultaneous Distillation Extraction (SDE) apparatus. The dichloromethane phase was heated in a 50 °C water bath, and the aqueous phase was heated in a 140 °C oil bath. Once a stable liquid-liquid interface was formed, extraction was carried out for 5 h. The organic phase was then collected and supplemented with 50 μL of 1.2028 mg/mL ethyl benzoate as the internal standard. The extract was concentrated to 2 mL using a rotary evaporator, dried over anhydrous sodium sulfate, filtered through a 0.22 μm organic phase membrane, and transferred to GC-MS vials for analysis.

#### 2.2.4 GC/MS determination

Neutral aroma compounds were analyzed using an Agilent 7890A gas chromatograph equipped with an HP-5MS capillary column (30 m × 0.25 mm i.d., 0.25 μm film thickness). Samples (1 μL) were injected in split mode with a 10:1 split ratio. Helium was used as the carrier gas at a flow rate of 1 mL/min. The temperature program was as follows: initial temperature of 40 °C held for 2 min, ramped at 2 °C/min to 200°C (held for 5 min), and then increased at 10 °C/min to 280 °C. Detection was performed using an Agilent 5975C mass spectrometer with a transfer line temperature of 250 °C and an ion source temperature of 230 °C under 70 eV electron impact ionization.

#### 2.2.5 Qualitative analysis of volatile aroma compounds

Chromatographic peaks obtained from GC-MS were matched to reference spectra using the NIST 14.0 mass spectral library (Agilent Technologies). The compound with the highest match was selected for identification. Quantification was performed using phenylethyl acetate as the internal standard. The concentration of each volatile compound was calculated using the formula: Wi = (30 × Mi)/(Si × 2/10), where Wi is the content of each volatile aroma compound (μg/g), Mi is the peak area of the compound, and Si is the peak area of the internal standard.

#### 2.2.6 Sensory quality evaluation

A portion of fermented tobacco was hand-rolled into cigars (14 mm in diameter, 110 mm in length). Sensory evaluation was conducted by trained experts from Enshi Cigar Tobacco Production Base using a nine-point scale (see Supplementary Table S1). The evaluation included eight indicators: aroma quality, aroma intensity, aftertaste, off-notes, sweetness, irritation, combustibility, and ash color. Each sample was evaluated in triplicate.

#### 2.2.7 Collection of surface microorganisms from cigar filler leaves

Processed tobacco samples were immersed in 80 mL of sterile distilled water at room temperature for 15 min. The suspension was subjected to 10 s of centrifugation, followed by 90 s of vortex mixing. The mixture was centrifuged at 500 × g for 1 min to remove leaf debris, then at 10,000 × g for 10 min to collect microbial cells. The pellet was washed with a buffer containing 100 mmol/L Tris-HCl, 50 mmol/L EDTA-Na_2_, 20 g/L PVP, 1 mL/L Tween-20, and 1.4 mol/L NaCl (pH 8.0). The mixture was rotated for 45 s at room temperature, incubated in a 65 °C water bath for 5 min, and centrifuged at 6,000 × g for 6 min. The washing steps were repeated until the supernatant became nearly colorless. The resulting microbial pellet (≥200 mg) was snap-frozen in liquid nitrogen for at least 20 min and stored at −80 °C.

#### 2.2.8 DNA extraction, library construction, and metagenomic sequencing

DNA extraction was performed using the CTAB method. Briefly, 1,000 μL of CTAB lysis buffer, lysozyme, and an appropriate amount of sample were added to a 2.0 mL EP tube and incubated in a 65 °C water bath with occasional inversion. The lysate was centrifuged, and the supernatant was extracted with phenol (pH 8.0):chloroform:isoamyl alcohol (25:24:1) and then with chloroform:isoamyl alcohol (24:1). After centrifugation, the supernatant was mixed with isopropanol and precipitated at −20 °C. The pellet was washed twice with 75% ethanol, air-dried, and dissolved in ddH_2_O. RNase A (1 μL) was added to remove RNA, followed by incubation at 37 °C for 15 min.

Library preparation and sequencing were performed by Beijing Novogene Co., Ltd. DNA samples were fragmented, end-polished, A-tailed, and ligated with Illumina adapters, followed by size selection. PCR amplification (except in PCR-free protocols) and purification were performed using the AMPure XP system. Libraries were quality-checked using the Agilent Fragment Analyzer and quantified using Qubit and qPCR. Qualified libraries were pooled and sequenced on Illumina platforms.

#### 2.2.9 Data analysis

Differences in microbial taxa before and after fermentation were analyzed using the cloud-based platforms provided by Beijing Novogene (https://magic.novogene.com/) and CNSknowall (https://cnsknowall.com). Raw sequencing data were processed using Fastp to obtain clean reads. Functional and taxonomic annotation was conducted using DIAMOND. Statistical significance was assessed using Statistica 23.0 software (SPSS (Statistical Package for the Social Sciences) Inc., Chicago, IL, United States of America, https://spss.updatestar.com).

## 3 Results and discussion

### 3.1 Changes in volatile aroma compounds during cigar filler leaves fermentation

**FIGURE 1:**
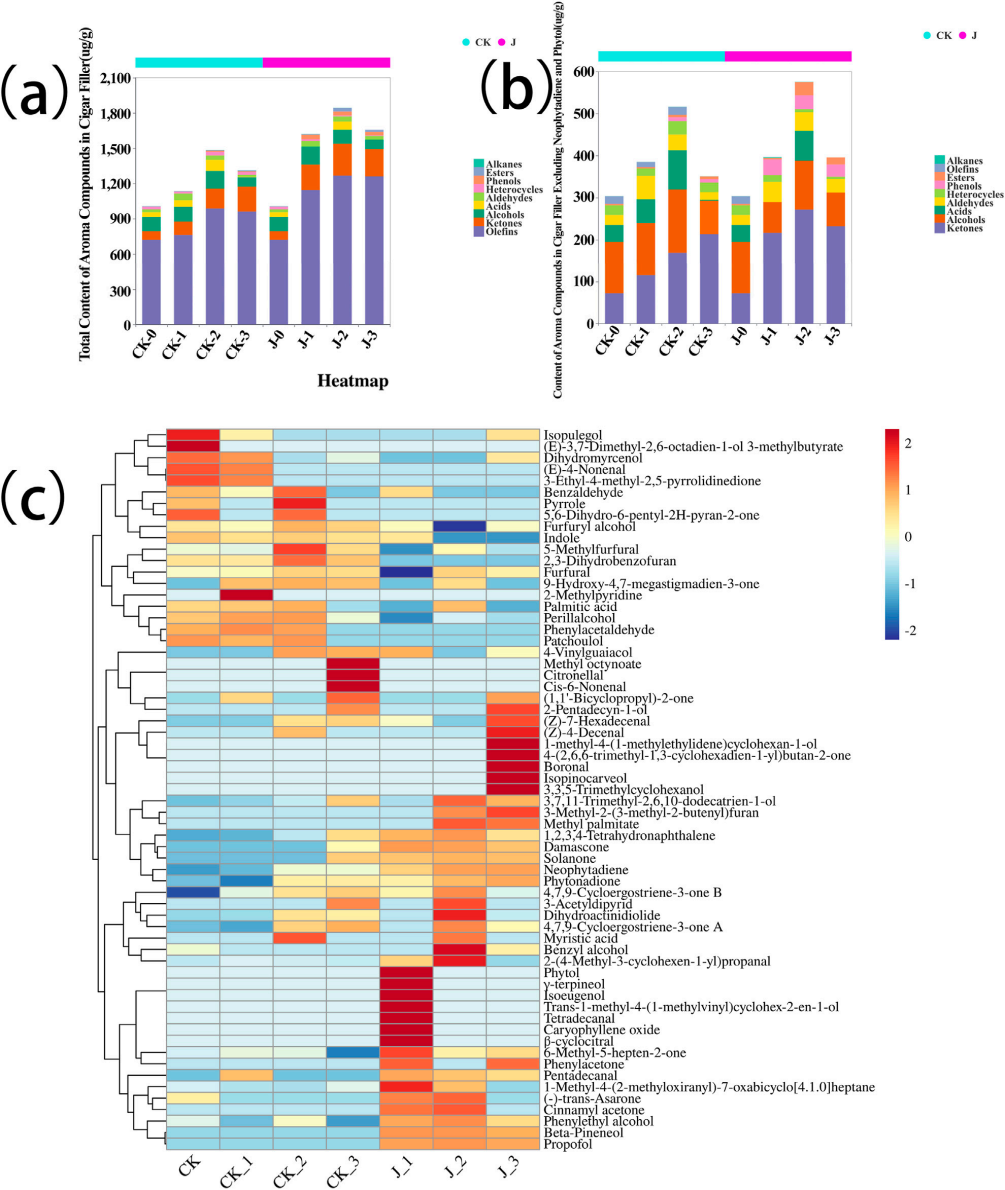
**(a)** Total content of volatile aroma compounds during cigar filler leaved, **(b)** Content of volatile aroma compounds in Cigar filler leaves except Neophytadiene and Phytol, **(c)** Heatmap of Aroma Compound Content in Cigar filler Leaves During Fermentation. Before fermentation (CK-0, J-0), after the first overturn (CK-1, J-1), after the second overturn (CK-2, J-2), and after the third overturn (CK-3, J-3).

Dynamic changes in the total aroma components of cigar tobacco leaves during fermentation are shown in Figure 1a, indicates an initial increase followed by a subsequent decline as fermentation progressed. These findings align with previous studies that reported similar dynamic trends in the fermentation of cigar tobacco leaves, where alcohols, alkenes, and ketones initially increased and then declined, while heterocyclic compounds exhibited an opposite trend (Hu, 2023). The heatmap in Figure 1c illustrates the distribution of individual aroma compounds, showing that notable substances such as damascone, solanone, and pentadecanal were present at significantly higher concentrations in the experimental group. These findings indicate that fermentation with *Staphylococcus saprophyticus* effectively enhances the accumulation of aroma-active compounds in cigar filler leaves, potentially enhancing their unique flavor profile. Sensory quality evaluation further confirmed that the inoculated group showed substantial improvements in aroma quality, aftertaste, and off-note suppression, along with increased aroma intensity and sweetness, and a marked reduction in irritation (see Supplementary Table S2). For detailed classification and quantification of the volatile aroma compounds, refer to Supplementary Figure S1.

After excluding neophytadiene and phytol from the analysis (Figure 1b), the overall trend in the total content of volatile aroma compounds remained consistent: concentrations first increased and then decreased. These findings underscore the critical role of *S. saprophyticus* in regulating the production and transformation of aroma compounds during fermentation. Moreover, the increased diversity of aroma compounds in the inoculated group may have made a significant contribution to the development of the characteristic flavor profile of cigar filler tobacco. For further details, see Supplementary Figure S2.

These results demonstrate that exogenous inoculation with *Staphylococcus saprophyticus* not only enhances aroma diversity and intensity but also accelerates the overall aroma development process. Notably, the inoculated group reached a total aroma content of 1,619 μg/g as early as the first overturn (day 7, J-1), surpassing the control group’s peak of 1,484 μg/g observed at the second overturn (day 14, CK-2). This earlier peak suggests that the fermentation period could be shortened by approximately 7 days without compromising aroma quality, presenting a valuable opportunity for process optimization.

From an industrial perspective, a shortened fermentation cycle offers multiple advantages: (i) enhanced warehouse turnover by enabling more production batches per year; (ii) reduced energy and labor costs due to decreased demands for environmental control and manual handling; and (iii) improved batch consistency, as the use of a defined starter culture may reduce reliance on operator experience and minimize inter-batch variation. It should be noted, however, that although the aroma peak occurs earlier, the subsequent aging phase remains essential for leaf color development, tar release, and flavor refinement. Future research should therefore integrate fermentation kinetics with sensory evaluation to minimize the minimum duration required to achieve stable, commercial-grade quality. Once validated at scale, a fermentation protocol utilizing *S. saprophyticus* could offer a novel, cost-effective strategy for the cigar industry.

### 3.2 Changes in microbial community structure and their impact on the quality of cigar filler leaves

#### 3.2.1 OTU variation analysis and α-diversity

**FIGURE 2:**
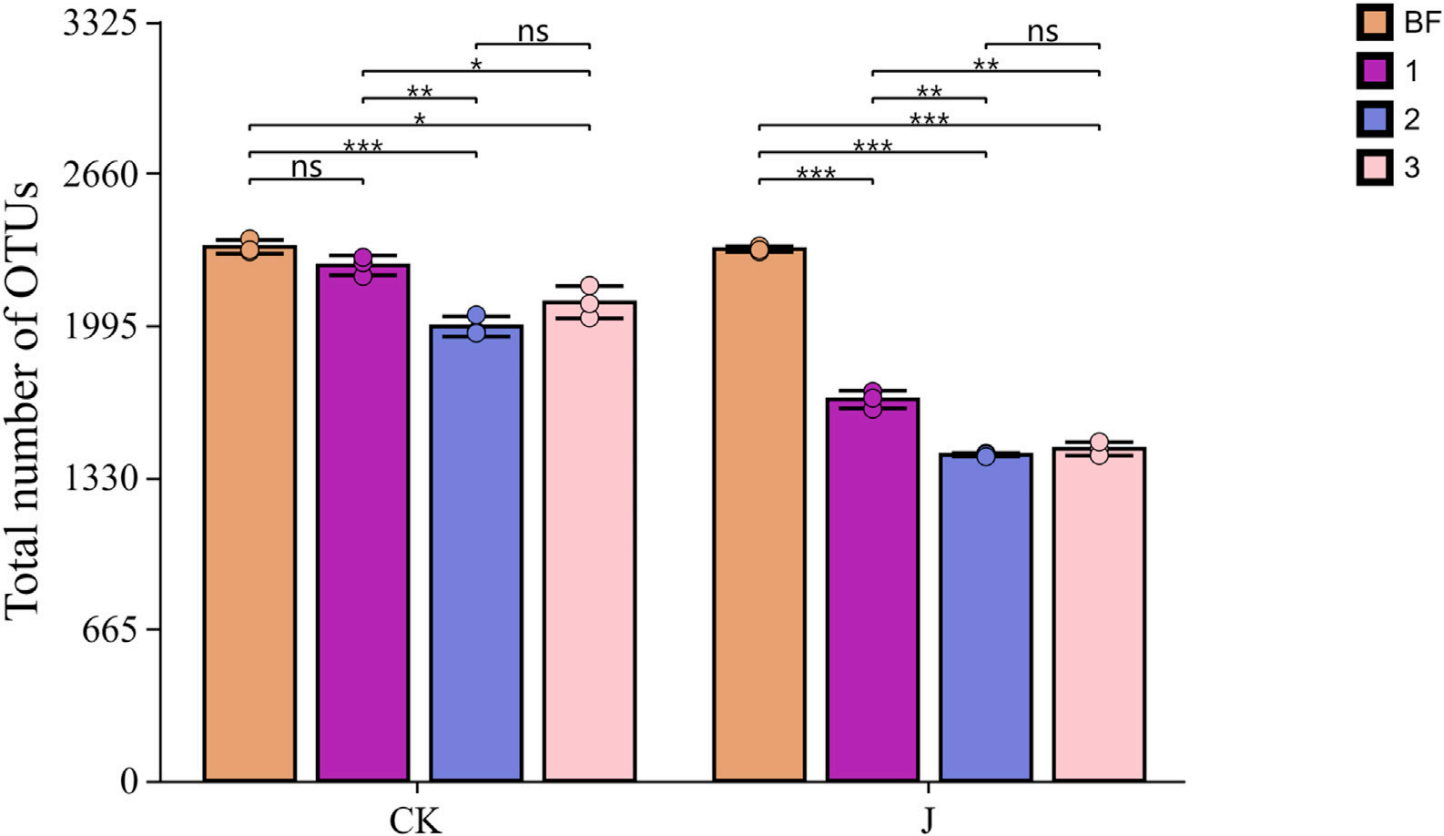
Analysis of OTU Count Differences in Cigar Filler Leaves under Various Treatments. CK denotes the natural fermentation group, and J denotes the inoculated fermentation group; BF indicates the sample before fermentation, “1” denotes the sample after the first turning, “2” refers to the sample after the second turning, and “3” refers to the sample after the third turning. Asterisks and horizontal lines above the bars indicate statistically significant differences between groups. * indicates p < 0.05, ** indicates p < 0.01, *** indicates p < 0.001, while “ns” denotes no significant difference.

The distribution of the microbial community composition at the OTU level on the surface of cigar filler tobacco leaves is shown in Figure 2. Before fermentation (CK-0), the surface OTU count reached its maximum at 2,374. After three overturns, the number of OTUs declined to 2,283, 1,965, and 2,152, respectively for natural fermentation. In contrast, the inoculated fermentation group (J-1, J-2, J-3) exhibited a more pronounced decline, with OTU counts decreasing to 1,667, 1,425, and 1,461, respectively.

**FIGURE 3:**
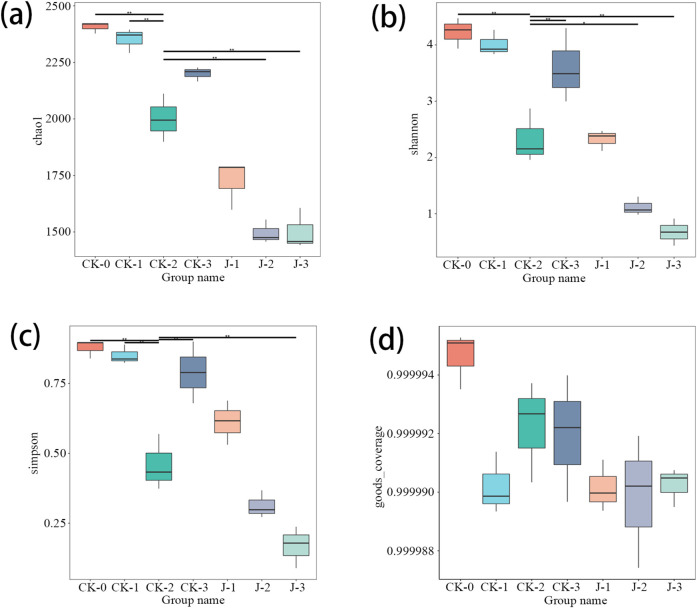
The α-diversity indices of bacteria in cigar filler tobacco leaves. **(a)** Chao1 Index, **(b)** Shannon Index, **(c)** Simpson Index, **(d)** Coverage Rate. Before fermentation (CK-0, J-0), after the first overturn (CK-1, J-1), after the second overturn (CK-2, J-2), and after the third overturn (CK-3, J-3).

The results of α-diversity indices in Figure 3 showed that, by the third overturn, both Chao1 and Shannon indices were significantly reduced in the *Staphylococcus saprophyticus*-inoculated group, indicating a decline in microbial richness and diversity. This reduction may be attributed to the ecological dominance of *S. saprophyticus*, which likely outcompeted and inhibited the growth of other microbial taxa (Sosa-Fajardo et al., 2024). Similar observations have been reported in previous studies, where the inoculation of microorganisms such as *Acinetobacter* sp. 1H8 and *A. indicus* 3B2 led to decreased Chao1 and Shannon indices (Zheng et al., 2022), and the addition of *Bacillus velezensis* was also associated with a reduction in Shannon indices compared to natural fermentation (Zhang, 2023).

Moreover, a reduction in the number of bacterial OTUs in the natural fermentation group relative to the pre-fermentation stage was found, particularly after the second overturn (p < 0.001) and third overturn (p < 0.05). This finding contrasts with earlier reports, which documented an increase in OTU abundance during fermentation (Yao et al., 2024). This discrepancy may be driven by the combined effects of several ecological mechanisms, including resource depletion, microbial antagonism, and environmental selection pressure. During fermentation, dominant taxa may rapidly consume the limited nutrients available in the system, thereby constraining the growth and expansion of other microbial populations (Nwogwu, 2022). Concurrently, certain strains may secrete antimicrobial metabolites—such as organic acids, hydrogen peroxide, or bacteriocins—that further inhibit the proliferation of potential competitors. Microorganisms with strong adaptability to evolving fermentation conditions are more likely to persist through natural selection, whereas less adaptable strains are gradually eliminated from the community (Sudiartha et al., 2023; Xing et al., 2021). Moreover, under dual pressures of nutrient scarcity and environmental stress, genetic drift may also occur within the microbial population, resulting in a stochastic decline in overall community diversity (Wani et al., 2022; Lynch et al., 2016).

Mechanistically, the early metabolic activity of *Staphylococcus saprophyticus* may have accelerated the depletion of available nutrients in the system while promoting the accumulation of fermentation-derived byproducts such as organic acids, alcohols, and reactive oxygen species. These metabolites can significantly alter the physicochemical properties of the fermentation environment, for instance by reducing pH, increasing osmotic pressure, and disrupting redox balance, thereby suppressing the growth and metabolic activity of microorganisms with lower environmental adaptability (Guan and Liu, 2020). This ecological filtering effect favors the survival of nutrient-tolerant strains and ultimately contributes to a contraction in overall community diversity.

As fermentation progressed, particularly during the third overturn, environmental changes such as enhanced oxygen diffusion may have supported the partial recovery of facultative aerobic or subdominant microbial taxa. Increased oxygen availability can alleviate anaerobic stress and facilitate more efficient energy production via oxidative phosphorylation, thus improving microbial competitiveness under nutrient-limited conditions (Li et al., 2020; Yang et al., 2024). Nevertheless, despite this partial rebound, the sustained ecological dominance of *S. saprophyticus*—through continued resource competition, production of inhibitory metabolites, and stable niche occupation—likely constrained the re-establishment of a diverse microbial community, resulting in persistently lower microbial diversity compared to the natural fermentation group.

#### 3.2.2 Alterations in the bacterial community structure of cigar filler leaves under different treatments

**FIGURE 4:**
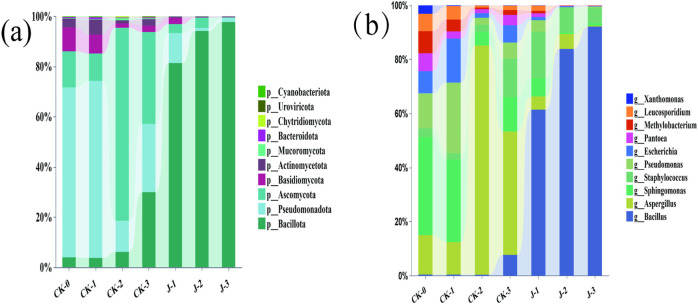
**(a)** Microbial community composition of cigar filler leaves at the phylum levels, **(b)** Microbial community composition of cigar filler leaves at the genus levels. Before fermentation (CK-0, J-0), after the first overturn (CK-1, J-1), after the second overturn (CK-2, J-2), and after the third overturn (CK-3, J-3).

The growth and metabolic activity of microbial communities are critical determinants of cigar tobacco leaves (Huang et al., 2021). The microbial community of cigar filler leaves was primarily composed of four phyla (Figure 4a): *Bacillota, Pseudomonadota, Ascomycota*, and *Basidiomycota*.

At the genus level, the microbial community of cigar filler leaves was dominated by four genera (Figure 4b): *Bacillus*, *Staphylococcus*, *Aspergillus*, and *Sphingomonas*. In the non-fermented group, *Sphingomonas* exhibited the highest relative abundance, whereas *Staphylococcus* and *Bacillus* were less prevalent. During natural fermentation, *Sphingomonas* showed a decreasing trend initially, followed by a resurgence, while *Aspergillus* increased significantly, peaking toward the end of fermentation. The relative abundances of *Staphylococcus* and *Bacillus* gradually increased over time.

In the *S. saprophyticus*-inoculated group, *Bacillus* showed extensive proliferation, with its relative abundance exceeding 92% at the end of fermentation and peaking at all three turnover points. *Staphylococcus* reached its maximum abundance after the first overturn and gradually declined thereafter, a trend mirrored by *Sphingomonas* and *Aspergillus*. These shifts indicate that the introduction of *S. saprophyticus* significantly reshaped the microbial ecosystem on the surface of the cigar filler leaves, accelerating microbial succession and altering both the composition and diversity of the microbial community. LEfSe analysis results were shown in Supplementary Figure S3.

As one of the key bacterial taxa in tobacco fermentation, *Bacillus* has been shown in numerous studies to play a pivotal role in enhancing tobacco quality. For example, *Bacillus* cereus has been reported to degrade hemicellulose and cellulose, improving the physical properties of wrapper leaves (LI, 2022). Dominant bacteria in cigar leaves are also capable of degrading aromatic hydrocarbons, aliphatic compounds, and lignin (Liu et al., 2021). Moreover, *Bacillus* species can hydrolyze tobacco proteins, thereby reducing bitterness and astringency (Chen Xing, 2015). Other species such as *Rhodococcus*, *Bacillus*, and *Pseudomonas* are effective in degrading nicotine (Zhong et al., 2010; Liu et al., 2015). Additionally, *Bacillus* has been found to break down starch (Pen et al., 1992), cellulose (Li et al., 2006), and other macromolecular substances, thereby reducing harshness. Collectively, these findings highlight the functional versatility of *Bacillus* in decomposing complex compounds in tobacco leaves, which improves fermentation efficiency and enhancing product quality.

#### 3.2.3 Predictive metabolic functions of microbiota

**FIGURE 5:**
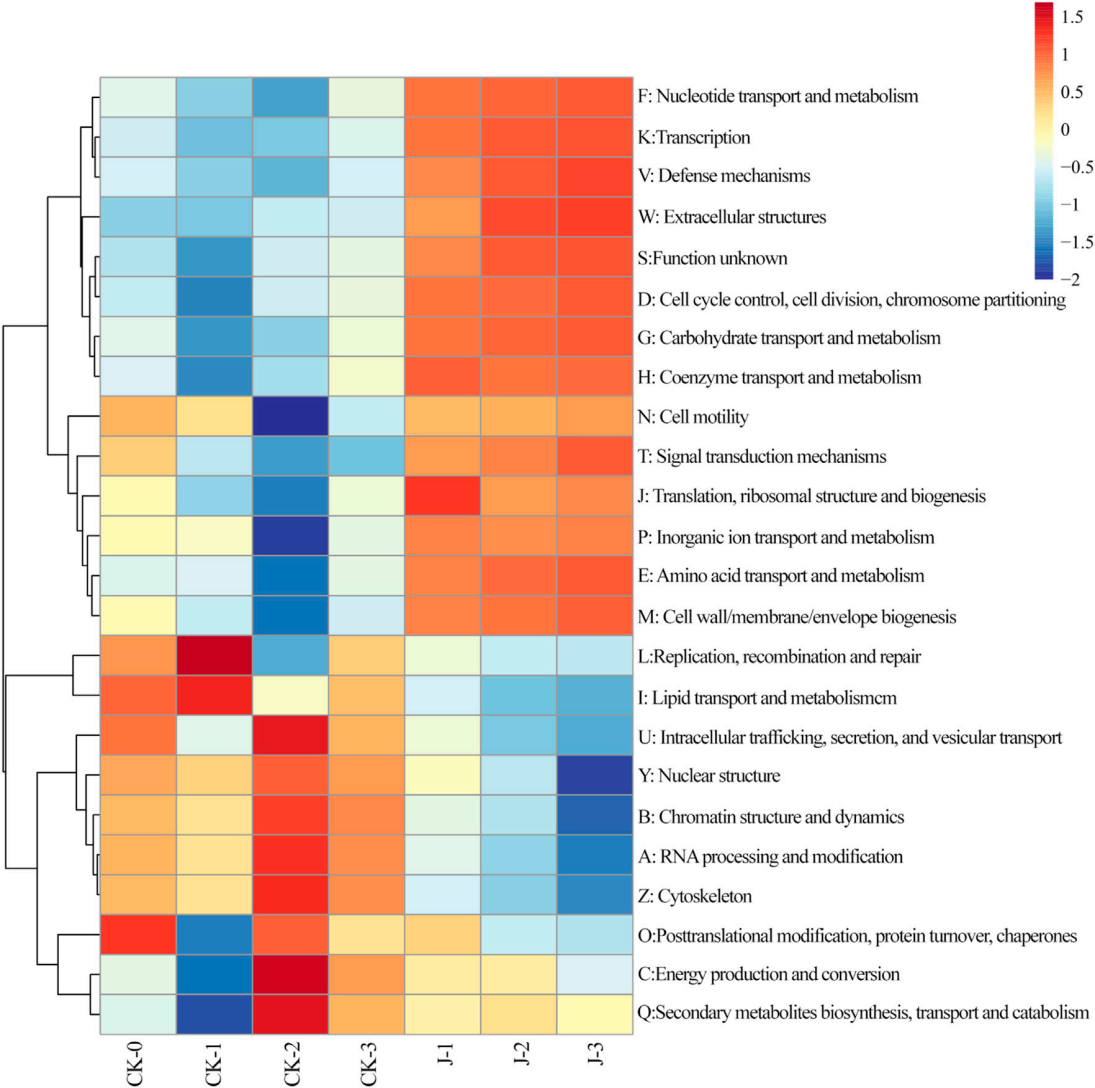
Heatmap of evolutionary genealogy of genes: non-supervised orthologous groups (EggNOG) Level 1 in cigar filler tobacco leaves. The values in the heatmap correspond to the Z-scores obtained after normalizing the relative abundance of each row. Before fermentation (CK-0, J-0), after the first overturn (CK-1, J-1), after the second overturn (CK-2, J-2), and after the third overturn (CK-3, J-3).

According to KEGG metabolic pathway analysis, the five most abundant functional categories were carbohydrate metabolism, amino acid metabolism, membrane transport, energy metabolism, and metabolism of cofactors and vitamins, consistent with previous findings (Yao et al., 2024). For further details, refer to Supplementary Figure S4. Functional gene annotation results conducted using the EggNOG database was shown in Figure 5.

Notably, the significant enrichment of genes involved in nucleotide transport and metabolism suggests enhanced nucleic acid turnover during fermentation, including key processes such as DNA replication, RNA transcription, repair, and degradation (Lane and Fan, 2015). In metabolically active fermentation environments, microbial communities necessitate rapid genome replication to adapt to environmental stresses and sustain ecological competitiveness. Furthermore, accelerated synthesis of rRNA and tRNA likely enhances translational activity, thereby promoting the initiation and progression of various downstream secondary metabolic pathways. Consistently, the enrichment of genes related to transcription, translation, and ribosomal structure indicates a significant increase in microbial growth rates and protein biosynthesis during fermentation (Zang et al., 2024; Zhang et al., 2022). This indicates that during specific fermentation stages, the efficient synthesis of metabolic enzymes, transporters, and transcriptional regulators represents a core biological mechanism underpinning system-wide metabolic activity and transformation efficiency.

Moreover, the persistently high abundance of genes associated with carbohydrate and amino acid metabolism highlights the central importance of these pathways during fermentation, aligning with KEGG Level 2 functional categorization. Enhanced carbohydrate metabolism not only facilitates rapid energy production through glycolysis and the pentose phosphate pathway (PPP), but also supplies precursor substrates for the biosynthesis of lipids, alkaloids, and other secondary metabolites. Simultaneously, activated amino acid metabolism supports protein synthesis and regulates aroma precursor formation and microbial signaling, thereby playing a pivotal role in modulating fermentation dynamics (Guo et al., 2023).

To further explore functional contributions, the relationship between enriched microbial genes and EggNOG functional categories was analyzed in the *S. saprophyticus*-inoculated group (Supplementary Figure S5). Sankey diagram results showed that *Aspergillus* was associated with all 24 functional categories, followed by *Sphingomonas* (22), *Pseudomonas* (21), *Staphylococcus* (20), and *Bacillus* (19). These associations indicate that the introduction of *S. saprophyticus* not only enhanced gene enrichment in these genera but also significantly increased the abundance of genes in corresponding metabolic pathways. This functional enhancement likely improved metabolic efficiency, stimulated microbial activity, and influenced the final outcomes of fermentation and flavor formation. Functional gene annotation results based on the CAZy database are provided in the Supplementary Figure S6.

Overall, the persistently high levels of the glycosyltransferase, carbohydrate-binding module, and carbohydrate esterase genes in the experimental group likely facilitated the degradation and transformation of complex sugars during fermentation, significantly shaping the chemical composition and flavor attributes of the cigar filler leaves. These results align with previous findings demonstrating that dominant metabolic enzymes—such as glycoside hydrolases (GHs) and glycosyltransferases (GTs)—play critical roles in the natural fermentation of cigar tobacco (Wu et al., 2023).

#### 3.2.4 Interactions between exogenous and endogenous microorganisms

**FIGURE 6:**
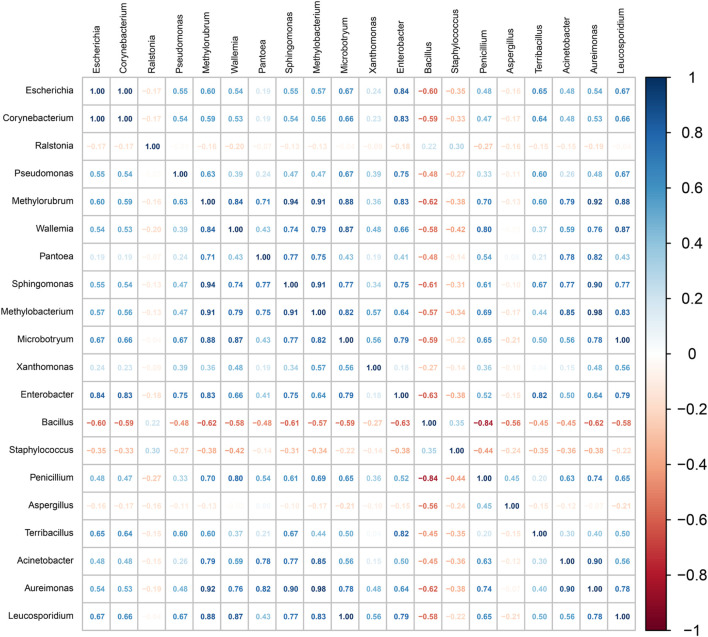
Heatmap of Spearman correlation coefficients based on representative bacterial groups. The correlation coefficient r ranges from −1 to 1. If r < 0, it indicates a negative correlation, and if r > 0, it indicates a positive correlation.

Microbial interactions are pivotal in shaping the community structure during fermentation processes (Mould and Hogan, 2021). As illustrated in Figure 6, a strong positive correlation was observed between *Staphylococcus* and *Bacillus*, both of which showed significant negative correlations with most of the other identified genera. This synergy may indicate cooperative nutrient utilization or metabolic exchange, facilitating mutual growth, while their antagonistic relationships with other taxa may stem from competition over resources or alteration of the local environment.

*Bacillus* and *Staphylococcus saprophyticus* are particularly noteworthy for their functional roles in the fermentation of cigar tobacco leaves. Both genera secrete a diverse array of extracellular enzymes—such as proteases, amylases, lipases, xylanases, and cellulases—that synergistically degrade macromolecular compounds including proteins, starches, lipids, and plant cell wall components. This enzymatic activity releases low-molecular-weight substrates that support microbial growth and promote tobacco maturation (Kumar et al., 2020; Yin et al., 2025). Although these two taxa may compete for initial abundant substrates (e.g., carbohydrates and proteins), their complementary enzymatic profiles facilitate more efficient decomposition of a wider range of compounds. For example, *S. saprophyticus*-derived xylanases and cellulases break down plant structural polymers, exposing additional carbon sources that benefit subsequent degraders like *Bacillus* (Si et al., 2023), thereby accelerating the overall fermentation process.

*Acinetobacter* exhibited a negative correlation with *Aquabacterium* but a positive correlation with *Bacillus*, suggesting both competitive and cooperative dynamics. Such patterns align with previous reports indicating strain-specific interactions—for instance, *Acinetobacter* indicus 3B2 inhibits *Aquabacterium* while promoting *Bacillus*, thereby influencing broader community dynamics involving *Staphylococcus* and *Aerococcus*. These interdependent relationships collectively drive microbial succession and impact fermentation outcomes.

#### 3.2.5 Correlation analysis between bacteria and key volatile flavor compounds

**FIGURE 7:**
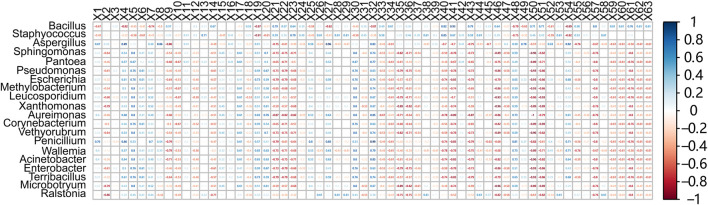
Correlation between bacterial communities and volatile flavor compounds. Heatmap analysis of representative bacteria and volatile flavor compounds (X1-X63) based on spearman correlation coefficients. The correlation coefficient r ranges from −1 to 1. If r < 0, it indicates a negative correlation, and if r > 0, it indicates a positive correlation. X1–X63 refer to the specific compound names: (1) Isopulegol, (2) (E)-3,7-Dimethyl-2,6-octadien-1-ol 3-methylbutyrate, (3) Dihydromyrcenol, (4) (E)-4-Nonenal, (5) 3-Ethyl-4-methyl-2,5-pyrrolidinedione, (6) Benzaldehyde, (7) Pyrrole, (8) 5,6-Dihydro-6-pentyl-2H-pyran-2-one, (9) Furfuryl alcohol, (10) Indole, (11) 5-Methylfurfural, (12) 2,3-Dihydrobenzofuran, (13) Furfural, (14) 9-Hydroxy-4,7-megastigmadien-3-one, (15) 2-Methylpyridine, (16) Palmitic acid, (17) Perillalcohol, (18) Phenylacetaldehyde, (19) Patchoulol, (20) 4-Vinylguaiacol, (21) Methyl octynoate, (22) Citronellal, (23) Cis-6-Nonenal, (24) (1,1'-Bicyclopropyl)-2-one, (25) 2-Pentadecyn-1-ol, (26) (Z)-7-Hexadecenal, (27) (Z)-4-Decenal, (28) 1-methyl-4-(1-methylethylidene)cyclohexan-1-ol, (29) 4-(2,6,6-trimethyl-1,3-cyclohexadien-1-yl)butan-2-one, (30) Boronal, (31) Isopinocarveol, (32) 3,3,5-Trimethylcyclohexanol, (33) 3,7,11-Trimethyl-2,6,10-dodecatrien-1-ol, (34) 3-Methyl-2-(3-methyl-2-butenyl)furan, (35) Methyl palmitate, (36) 1,2,3,4-Tetrahydronaphthalene, (37) Damascone, (38) Solanone, (39) Neophytadiene, (40) Phytonadione, (41) 4,7,9-Cycloergostriene-3-one B, (42) 3-Acetyldipyrid, (43) Dihydroactinidiolide, (44) 4,7,9-Cycloergostriene-3-one A, (45) Myristic acid, (46) Benzyl alcohol, (47) 2-(4-Methyl-3-cyclohexen-1-yl)propanal, (48) Phytol, (49) γ-Terpineol, (50) Isoeugenol, (51) Trans-1-methyl-4-(1-methylvinyl)cyclohex-2-en-1-ol, (52) Tetradecanal, (53) Caryophyllene oxide, (54) β-Cyclocitral, (55) 6-Methyl-5-hepten-2-one, (56) Phenylacetone, (57) Pentadecanal, (58) 1-Methyl-4-(2-methyloxiranyl)-7-oxabicyclo [4.1.0]heptane, (59) (-)-trans-Asarone, (60) Cinnamyl acetone, (61) Phenylethyl alcohol, (62) Beta-Pineneol, (63) Propofol.

Using Spearman correlation analysis was performed the relationships between 64 volatile compounds and 20 representative bacterial genera were analyzed. As shown in Figure 7, *Staphylococcus* exhibited positive correlations with 37 aromatic compounds, including 1-methyl-4-(2-methyloxiranyl)-7-oxabicyclo [4.1.0] heptane, cis-6-nonenal, and (Z)-7-hexadecenal, while showing negative correlations with 21 compounds such as 2-methylpyrazine, benzaldehyde, and isopulegol. *Bacillus* was positively correlated with 40 aroma compounds—including solanone, phenylethyl alcohol, damascone, and 6-methyl-5-hepten-2-one—and negatively correlated with 20 compounds, such as pyrrole, furfuryl alcohol, and (E)-4-nonenal. These findings highlight the central role of microbial metabolism in shaping the volatile flavor profile of cigar filler leaves.

*Bacillus* is a ubiquitous genus in natural environments, particularly in tobacco, where it plays a key role in fermentation by degrading macromolecules such as carotenoids into low-molecular-weight aromatic compounds. Previous studies have demonstrated the ability of *Bacillus* to enhance both the aroma and overall quality of tobacco products (English et al., 1967). For instance, fermentation with *B. velezensis* was reported to increase total aroma compounds in cigar leaves by 26.1%. Likewise, co-fermentation of *Bacillus megaterium* with yeast strains isolated from tobacco leaves led to a 56.4% increase in total volatile compounds, including elevated levels of solanone, further validating their contribution to aroma enhancement (Mao, 2023). In the present study, the solanone content in the inoculated group increased significantly to 67.4 μg/g, representing 170.7% increase.

Although studies on *Staphylococcus* in cigar fermentation remain limited, its role in meat fermentation provides valuable insights. In meat products, *Staphylococcus* species modulate microbial and biochemical dynamics, particularly processes involving proteins and fats, thereby influencing product quality and enhancing the formation of volatile flavor compounds (Li et al., 2015). For example, *Staphylococcus xylosus* has been shown to significantly increase the production of alcohols and esters in sausages, enriching the volatile flavor profile. Similarly, inoculation with *S. epidermidis*, *S. xylosus*, and *S. auricularis* into dry-cured meat led to the generation of distinct aroma compounds: *S. epidermidis* produced elevated levels of heterocyclic compounds such as 2,4,6-trimethylpyrimidine; *S. xylosus* enhanced the formation of aldehydes including 2-methylbutanal and 3-methylbutanal; and *S. auricularis* promoted the synthesis of esters like 6-deoxymannonic lactone.

In this study, the volatile compounds identified in cigar filler leaves were predominantly aldehydes and alcohols, which differed from those typically found in meat fermentations, likely due to the distinct substrate compositions. Meat is rich in proteins, fats, and free amino acids, whereas tobacco leaves primarily contain cellulose, polyphenols, carbohydrates, and alkaloids such as nicotine. These substrate differences lead to divergent microbial metabolic pathways, resulting in the production of distinct volatile compounds during fermentation. Despite these differences, the observed contribution of *Staphylococcus* to aroma formation in cigar fermentation highlights its potential for improving sensory quality, although further research is needed to elucidate its specific metabolic mechanisms.

## 4 Conclusion

This study comprehensively investigated the impact of *Staphylococcus saprophyticus* inoculation on aroma compound formation and microbial community structure during the fermentation of cigar filler leaves. The results demonstrate the functional potential of *S. saprophyticus* as an exogenous microorganism for improving the fermentation quality of cigar tobacco. *S. saprophyticus* enhances the production of characteristic aroma compounds, stimulates key metabolic pathways, and reshapes microbial community dynamics through interactions with native microorganisms. These combined effects provide a promising strategy for optimizing cigar fermentation processes and enhancing product sensory qualities. Additionally, this strain also shows potential for shortening the fermentation cycle, which may contribute to improved production efficiency and cost-effectiveness in medium-to large-scale cigar manufacturing systems.

## Data Availability

The datasets presented in this study can be found in online repositories. The names of the repository/repositories and accession number(s) can be found below: https://www.ncbi.nlm.nih.gov/, PRJNA1181943.
