# Supplementary material for: The impact of Staphylococcus saprophyticus on the fermentation of cigar filler tobacco leaves and the dynamics of microbial community
Source: Front Bioeng Biotechnol. 2025 Sep 23;13:1666879. doi: 10.3389/fbioe.2025.1666879 (PMC12500701; doi:10.3389/fbioe.2025.1666879)
Supplement: Supplementary file 1 [file Supplementaryfile1.docx]

Supplementary Material

The impact of *Staphylococcus saprophyticus* on the fermentation of cigar filler tobacco leaves and the dynamics of microbial community

Lan Yao^a,1^, Zhongde Zhao ^a,1^, Linwei Li^1^, Jun Yu^2^, Jinpeng Yang^2*^, Chunlei Yang^2*^, Xiong Chen^1*^

1. Key Laboratory of Fermentation Engineering (Ministry of Education), Cooperative Innovation Center of Industrial Fermentation (Ministry of Education & Hubei Province), College of Life and Health Science, Hubei University of Technology, 28th of Nanli Road, Wuhan 430068, China;
2. Tobacco Research Institute of Hubei Province, Wuhan 430030, China

a. the two authors contribute equally to this work.

*Correspondence: yjp2022@126.com (JY), ycl193737@163.com (CY), cx163_qx@163.com (XC).

Postal addresses: Hubei University of Technology, 28th of Nanli Road, Hongshan District, Wuhan 430068, China

**Sensory Quality Evaluation**

The results in Table S2 showed that unfermented cigar tobacco leaves exhibit poor performance in sensory evaluations, with weak aroma quality, insufficient aroma volume, a lack of sweetness, moderate grayness, and an unsatisfactory aftertaste characterized by noticeable off-notes and irritability, failing to meet overall taste expectations. However, the introduction of *Staphylococcus saprophyticus* significantly enhances sensory qualities of cigar filler leaves. Fermented tobacco leaves score markedly higher in sensory evaluations than the control group. Specifically, aroma quality, aftertaste, and off-notes were improved, with increased aroma volume and sweetness, and a significant reduction in irritation. The findings suggest that fermentation with *Staphylococcus saprophyticus* effectively improves the flavor and overall sensory experience of cigar tobacco leaves.

**Types of Aroma-Enhancing Compounds in Cigar filler leaves During Fermentation**

As illustrated in Fig. S1,63 aroma-enhancing compounds were identified. These compounds are categorized into nine classes based on their chemical structure: alkenes, alcohols, ketones, aldehydes, acids, heterocyclic compounds, esters, phenols, and alkanes. Among them, alcohols account for the largest proportion at 27%, with 17 types identified, followed by aldehydes at 22%, with 14 types identified, and ketones at 21%, with 13 types identified. Furthermore, heterocyclic compounds make up 11%, with 7 types, esters account for 6%, with 4 types identified, phenols comprise 5%, with 3 types. Acids and aldehydes each account for 3%, with 2 types identified respectively. Alkanes comprise the smallest proportion at 2%, with only 1 type identified. These findings indicate that alcohol compounds are the primary contributors to the aroma of cigar filler leaves.

**Changes in Aroma-Enhancing Compounds During Cigar filler leaves Fermentation**

To gain a deeper understanding of the unique aromatic compounds in cigars at different treatments and stages, a visualization tool known as the UpSet plot was introduced. This tool effectively illustrates the intersections among multiple sets, assisting us in identifying aroma compounds present exclusively at specific stages.

As illustrated in Fig. S2, after the third overturn in the experimental group, the richest variety of aroma compounds were detected, identifying 35 different aromatic compounds. Throughout the fermentation process, only 12 specific aroma compounds remained consistently present, highlighting significant changes in aroma composition at different stages. These persistent compounds may play a crucial role in shaping the distinctive aroma of cigars. Notably, after the first overturn in the experimental group, eight unique aroma compounds were identified, including (Z)-7-hexadecenal, isoeugenol, β-cyclocitral, γ-terpineol, caryophyllene oxide, trans-1-methyl-4-(1-methylvinyl) cyclohex-2-en-1-ol, Tetradecanal, and phytol. These compounds conveyed flavors such as fatty, herbal, lilac, sweet, fresh mint, citrus musk, and grassy notes. In contrast, only five unique aroma compounds were detected after the third overturn in the experimental group, which were 1-methyl-4-(1-methylethylidene) cyclohexan-1-ol, 4-(2,6,6-trimethyl-1,3-cyclohexadien-1-yl) butan-2-one, Boronal, 3,3,5-trimethylcyclohexanol, and isopinocarveol, with flavor profiles including terpinolene clove, floral, nutty, minty, and woody warmth. These unique flavors might result from fermentation with *Staphylococcus saprophyticus*. In the control group, five unique aroma compounds were detected after the three overturns, whereas in the experimental group, this number doubled to 13. This finding strongly suggests that adding *Staphylococcus saprophyticus* can enhance the variety of aroma compounds, potentially contributing to the unique flavor profile of cigar filler leaves during fermentation. These unique aroma compounds confer distinctive flavor characteristics to the tobacco leaves, further validating the pivotal role of exogenous microorganisms and mediums in the aroma formation of cigar leaves.

**Microbial Differences Across Fermentation Treatments**

LEfSe analysis further identified significant microbial differences across treatments, as shown in Fig. S3. The evolutionary cladogram depicts microbial taxa from phylum to species across concentric circles, with colors representing significant differences among treatment groups. Across pre-fermentation (CK-0), natural fermentation (CK), and inoculated fermentation (J) groups, differentially abundant taxa were distributed across 5 phyla, 7 classes, 11 orders, 12 families, 20 genera, and 79 species. In the pre-fermentation group, significant taxa included the classes Alphaproteobacteria and Gammaproteobacteria, with notable genera such as Sphingomonas and Pseudomonas. In the natural fermentation group, Sordariomycetes was a key differential class, and *Aspergillus* was identified as a characteristic genus. In the inoculated group, *Bacilli* was significantly enriched, with *Bacillus* emerging as the dominant genus.

**Predictive Metabolic Functions of Microbiota**

The KEGG database serves as a tool for systematically analyzing biological metabolic pathways, integrating gene and genomic information to reveal their roles within higher-level functional contexts.1-3 The microbial community structure under different treatments underwent significant changes, further influencing the distribution and activity of microbial metabolic pathways. Fig. S4 illustrates that the top five pathways by relative abundance in KEGG metabolic functions include carbohydrate metabolism, amino acid metabolism, membrane transport, energy metabolism, and the metabolism of cofactors and vitamins. Significant differences in the relative abundance of major metabolic pathways were observed between the experimental and control groups, particularly in carbohydrate metabolism, amino acid metabolism, membrane transport, and the metabolism of cofactors and vitamins. The experimental group showed markedly higher relative abundances in these pathways across three turnover processes, indicating that the addition of *Staphylococcus saprophyticus* significantly enhanced the activity of these metabolic functions. The enhancement of carbohydrate and amino acid metabolism might accelerate the conversion of sugars and proteins in tobacco leaves, thereby affecting cigar flavor formation. The improvement in membrane transport pathways may enhance the material exchange capacity of the microbial community; an increase in the metabolism of cofactors and vitamins suggests a heightened demand for these substances during fermentation. As essential components of metabolic reactions, these substances support microbial growth and metabolic activity, potentially enhancing the overall flavor and quality of the tobacco leaves.

**Functional Gene Enrichment Analysis Based on EggNOG Annotation**

EggNOG is a functional annotation database that classifies genes based on evolutionary relationships and broad biological functions through unsupervised clustering. As shown in Fig. S5, the experimental group (J-1, J-2, J-3) exhibited significantly higher gene abundance across multiple functional categories throughout the fermentation process. These included nucleotide transport and metabolism (F), transcription (K), defense mechanisms (V), extracellular structures (W), unknown functions (S), cell cycle control and chromosome partitioning (D), carbohydrate transport and metabolism (G), coenzyme transport and metabolism (H), cell motility (N), signal transduction mechanisms (T), translation and ribosomal biogenesis (J), inorganic ion transport and metabolism (P), amino acid transport and metabolism (E), and cell wall/membrane/envelope biogenesis (M). These findings suggest that the addition of *Staphylococcus saprophyticus* substantially reshaped the functional gene profile of the cigar leaf microbiome, particularly by enhancing metabolic and cellular activity-related pathways.

**CAZy Annotation of Carbohydrate Metabolism-Related Enzymes**

The CAZy database offers detailed classification and functional annotation of carbohydrate-active enzymes, which play essential roles in processes such as glycoside hydrolysis and glycosyl transfer, directly impacting microbial carbohydrate metabolism and fermentation functionality. Unlike KEGG and EggNOG, which provide broader functional overviews, CAZy focuses specifically on microbial enzymatic activities related to carbohydrate conversion.

In this study, CAZy analysis revealed significant differences in the abundance of glycosyltransferases (GTs), carbohydrate-binding modules (CBMs), and carbohydrate esterases (CEs) between the experimental (J-1, J-2, J-3) and control (CK-1, CK-2, CK-3) groups (Fig. S6). Throughout fermentation, the gene abundance of GTs and CBMs in the experimental group remained consistently higher than in the control. Notably, CBM abundance increased with fermentation progression, peaking during the third overturn, suggesting enhanced microbial capability to bind complex carbohydrates at later stages. Similarly, CE gene abundance was higher in the experimental group during the first and third overturns, implying that *S. saprophyticus* may facilitate ester bond hydrolysis in carbohydrate structures during the early and late stages of fermentation. Although CE abundance was higher in the control group during the second overturn, this may reflect stage-specific enzymatic responses or transient microbial shifts.

**References**

Kanehisa, M., Furumichi, M., Sato, Y., Matsuura, Y. and Ishiguro-Watanabe, M.; KEGG: biological systems database as a model of the real world. Nucleic Acids Res. 53, D672-D677 (2025).

Kanehisa, M; Toward understanding the origin and evolution of cellular organisms. Protein Sci. 28, 1947-1951 (2019)

Kanehisa, M. and Goto, S.; KEGG: Kyoto Encyclopedia of Genes and Genomes. Nucleic Acids Res. 28, 27-30 (2000).

**Figure Legends**

**Fig. S1:** Proportion of Aroma-Enhancing Compound Types in Cigar filler During Fermentation.

**Fig. S2:** UpSet Plot of Aroma Compound Varieties in Cigar filler.

**Fig. S3:** Differential Microbes in Cigar filler leaves with Various Treatments. CK denotes the natural fermentation group, CK-0 indicates the pre-fermentation samples, and J represents the inoculated fermentation group.

**Fig. S4:** KO Pathway Level 2 Analysis Diagram.

**Fig. S5:** Heatmap of Evolutionary Genealogy of Genes: Non-supervised Orthologous Groups (EggNOG) Level 1 in cigar filler tobacco leaves. The values in the heatmap correspond to the Z-scores obtained after normalizing the relative abundance of each row.Before fermentation (CK-0, J-0), after the first overturn (CK-1, J-1), after the second overturn (CK-2, J-2), and after the third overturn (CK-3, J-3).

**Fig. S6:** Heatmap of Carbohydrate-Active enZymes (CAZy) Level 1 in cigar filler tobacco leaves. The values in the heatmap correspond to the Z-scores obtained after normalizing the relative abundance of each row.Before fermentation (CK-0, J-0), after the first overturn (CK-1, J-1), after the second overturn (CK-2, J-2), and after the third overturn (CK-3, J-3).

**Table Legends**

**Table S1:** Sensory Quality Evaluation Standards for Cigar Tobacco Leaves

**Table S2:** Sensory Evaluation Scores of Cigar Tobacco Leaf Samples

**Tables**

**Table S1**

| **Indicators** | **Characteristics** | **Score** | **Indicators** | **Characteristics** | **Score** |
| --- | --- | --- | --- | --- | --- |
|  | Good, Better, Excellent | 7~9 |  | Notable, Remarkable, Highly Notable | 7~9 |
| Aroma quality | Slightly Poor, Average, Fair | 4~6 | Sweetness | Slightly Noticeable, Moderate, Fairly Noticeable | 4~6 |
|  | Very Poor, Poor, Below Average | 1~3 |  | None, Minimal, Barely Noticeable | 1~3 |
|  | Adequate, Sufficient, Abundant | 7~9 |  | Very Small, Small, Quite Small | 7~9 |
| Aroma volume | Slight Presence, Moderate, Satisfactory | 4~6 | Irritation | Slightly Small, Medium, Slightly Large | 4~6 |
|  | Scarce, Limited, Minimal | 1~3 |  | Considerably Large, Large, Very Large | 1~3 |
|  | Comfortable, Pleasant, Very Comfortable | 7~9 |  | Excellent, Good, Quite Good | 7~9 |
| Aftertaste | Slightly Comfortable, Moderate, Fairly Comfortable | 4~6 | Combustibility | Somewhat Better, Moderate, Slightly Worse | 4~6 |
|  | Very Poor, Poor, Unsatisfactory | 1~3 |  | Rather Poor, Poor, Stalled | 1~3 |
|  | Tiny, Small, Modest | 7~9 |  | Pale, White, Very White | 7~9 |
| Off-notes | Slightly Small, Medium, Somewhat Large | 4~6 | Ash color | Gray, Neutral, Grayish White | 4~6 |
|  | Considerable, Large, Vast | 1~3 |  | Black, Dark Gray, Grayish Black | 1~3 |

**Table S2**

| **Treatment** | **Sample** | **Individual Sensory Score（0~9）** | | | | | | | | **Overall Score** |
| --- | --- | --- | --- | --- | --- | --- | --- | --- | --- | --- |
|  |  | **Aroma quality,** | **Aroma volume** | **Aftertaste** | **Off-notes** | **Sweetness** | **Irritation** | **Combustibility** | **Ash color** |  |
| Control Group  CK | CK-0 | 5 | 5 | 6 | 4 | 4 | 6 | 6 | 5 | 41 |
|  | CK-1 | 5 | 5 | 5 | 6 | 4 | 6 | 6 | 5 | 42 |
|  | CK-2 | 6 | 5 | 5 | 6 | 5 | 7 | 6 | 5 | 45 |
|  | CK-3 | 6 | 6 | 6 | 6 | 6 | 7 | 6 | 6 | 49 |
| Experimental Group  J | J-0 | 5 | 5 | 6 | 4 | 4 | 6 | 6 | 5 | 41 |
|  | J-1 | 5 | 5 | 5 | 6 | 5 | 6 | 6 | 5 | 43 |
|  | J-2 | 7 | 6 | 5 | 6 | 6 | 7 | 6 | 5 | 48 |
|  | J-3 | 6 | 7 | 6 | 6 | 6 | 7 | 6 | 6 | 50 |

**Figures**


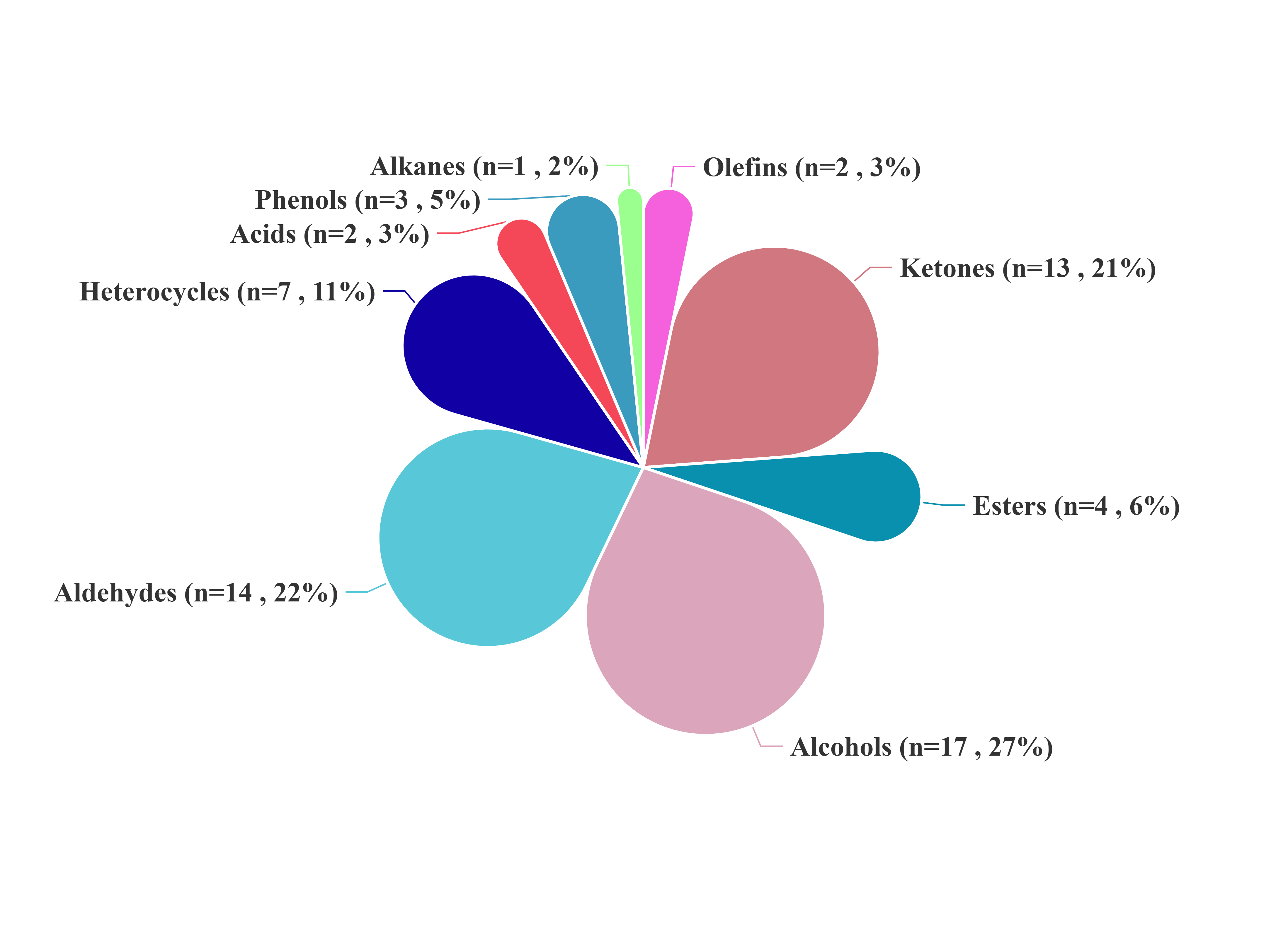


**Fig. S1**


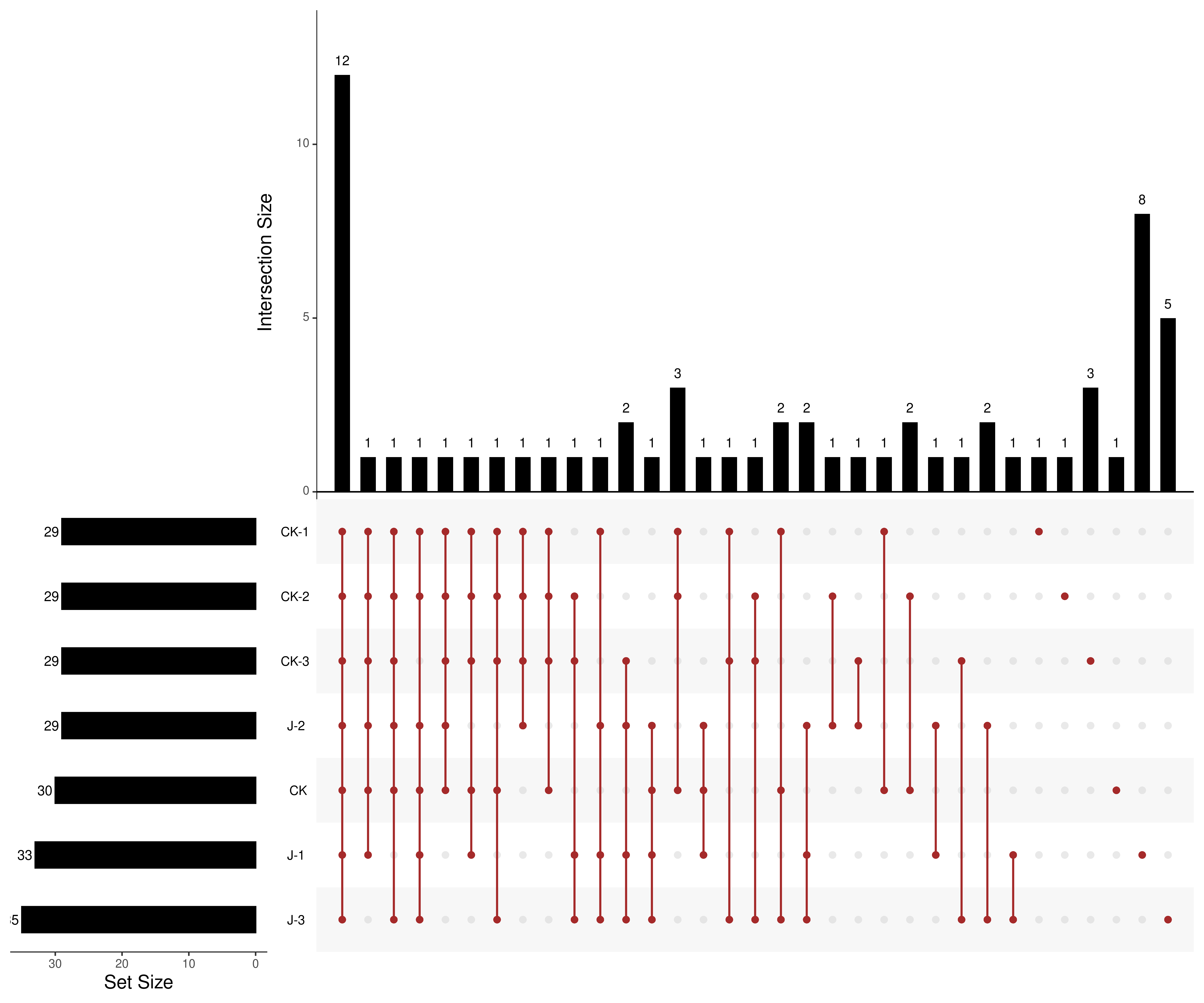


**Fig. S2**


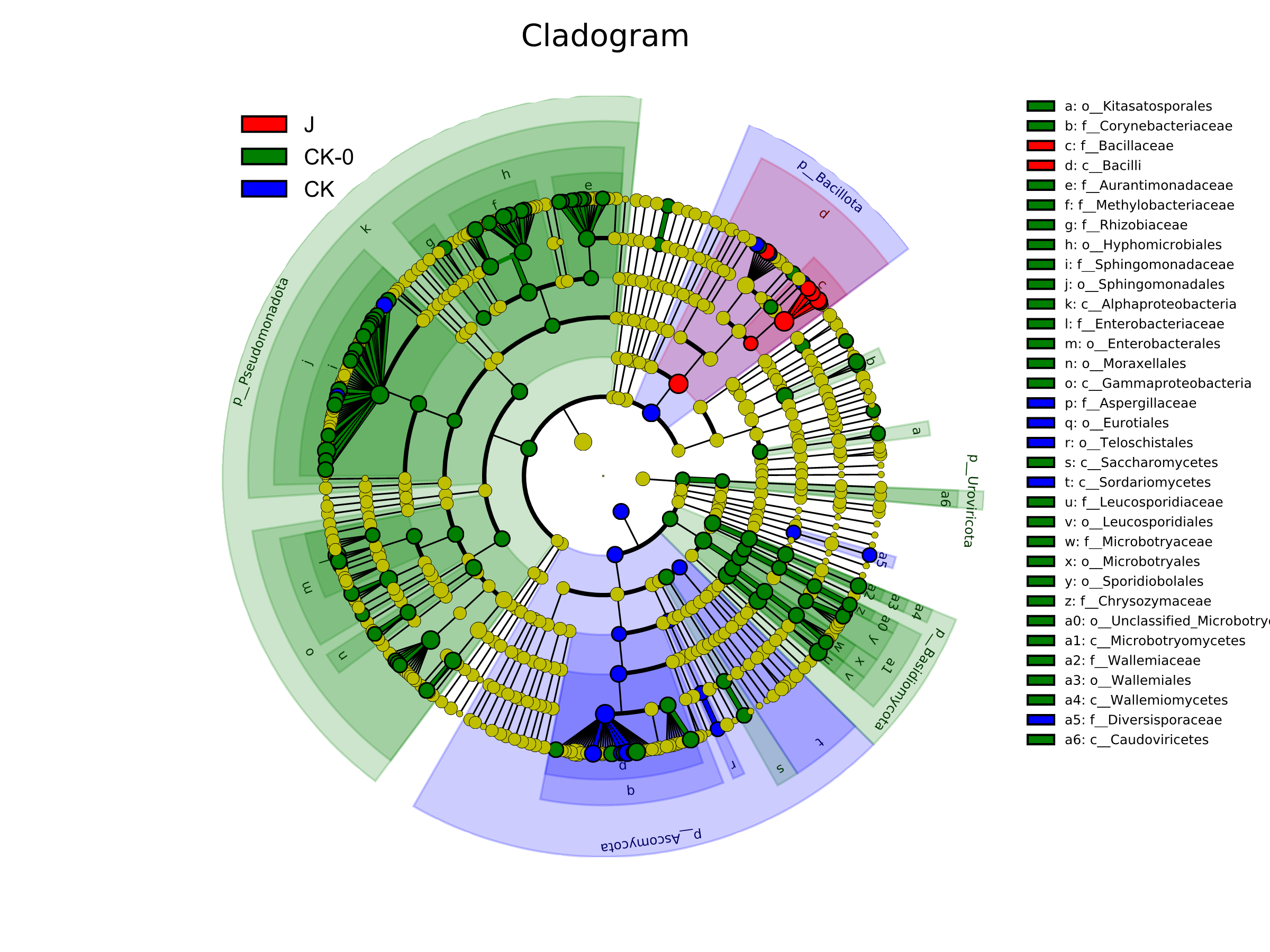


**Fig. S3**


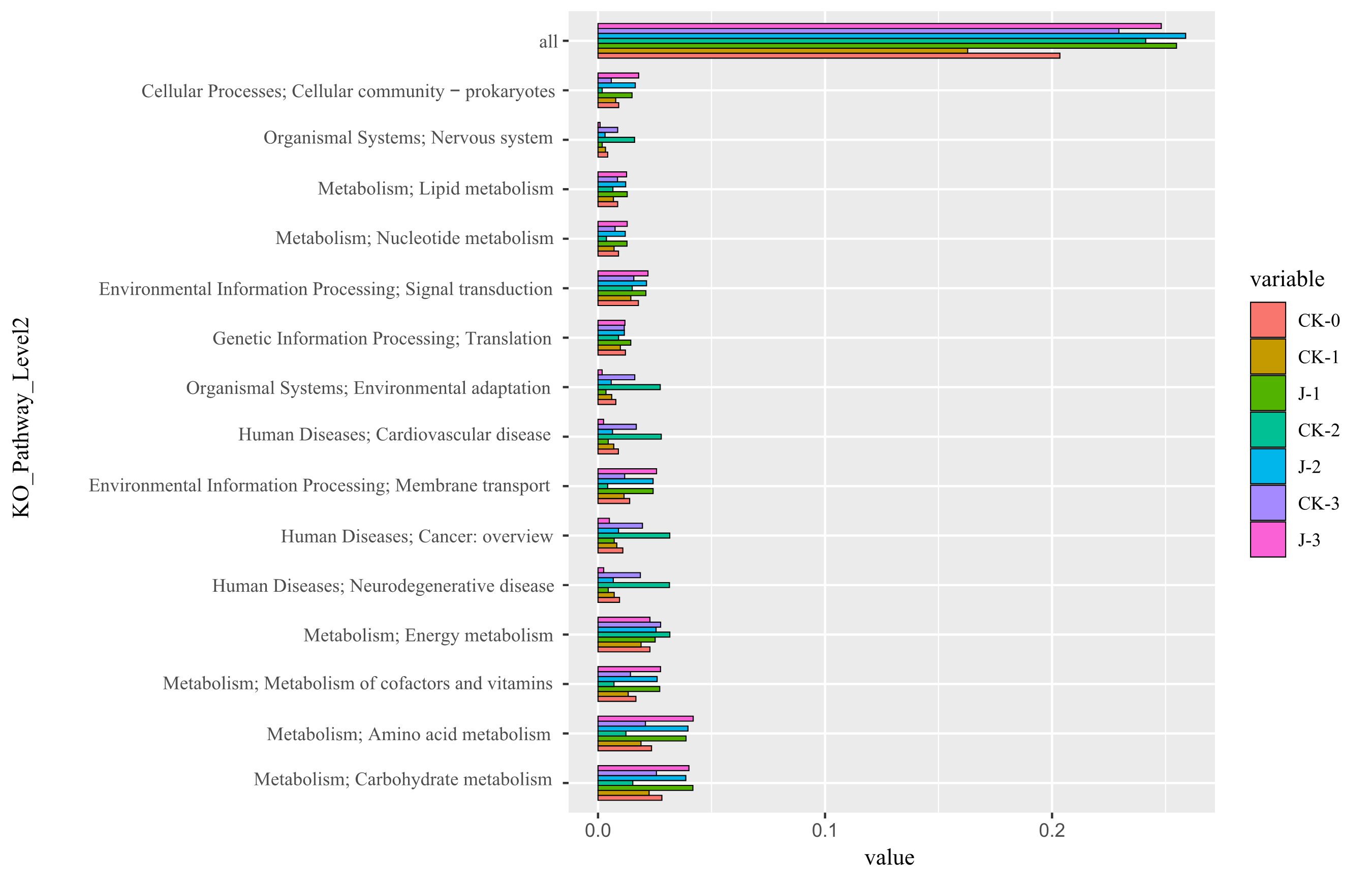


**Fig. S4**


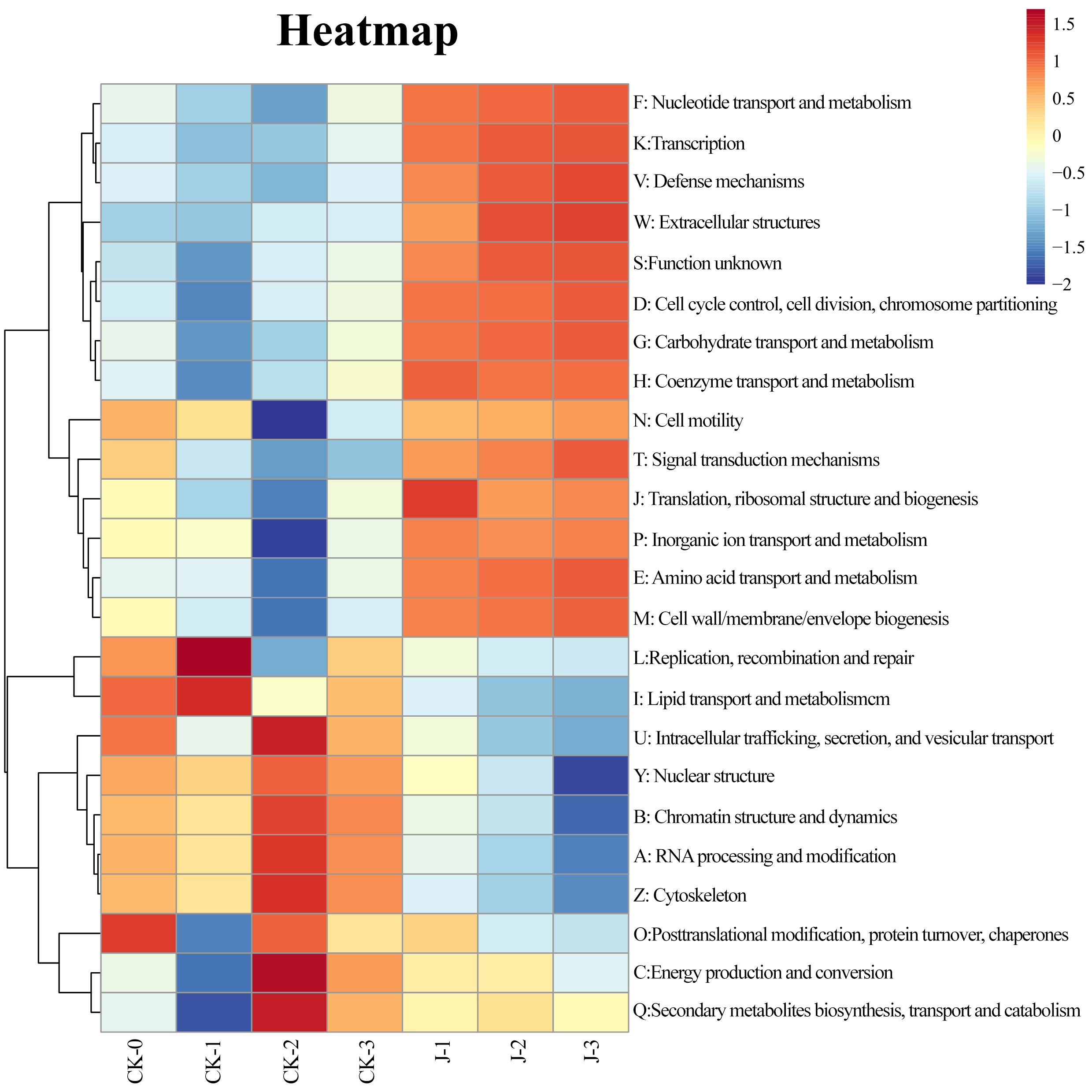


**Fig. S5**


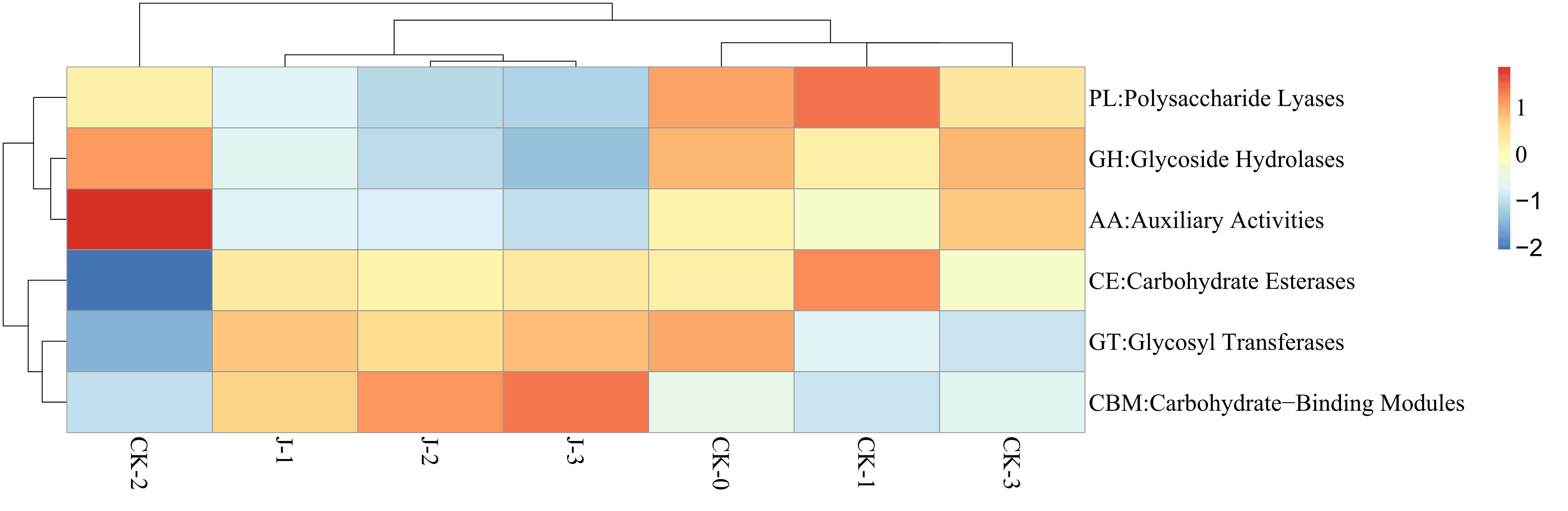


**Fig. S6**
